# Survey of Mymarommatidae and their occurrence in agricultural systems in Brazil

**DOI:** 10.1093/jis/14.1.15

**Published:** 2014-01-01

**Authors:** Vera Lúcia Rodrigues Machado Benassi, Fabrício Iglesias Valente, Jessica Cristina Lenzi, Simão Carvalho

**Affiliations:** 1 Instituto Capixaba de Pesquisa, Assistência Técnica e Extensão Rural – INCAPER, Rod. BR 101, Km 151, Linhares, Espírito Santo, Brazil; 2 Scholarship in Technical Support to Research - Conselho Nacional de Desenvolvimento Científico e Tecnológico – CNPq, Brazli

**Keywords:** Hymenoptera, parasitoid, yellow pan traps

## Abstract

Mymarommatidae surveys were carried out through the use of yellow pan traps in crops of green dwarf coconut, *Cocos nucifera* L. (Arecales: Arecaceae), papaya, *Carica papaya* L. (Brassicales: Caricaceae), citrus, *Citrus* spp. L. (Sapindales: Rutaceae), and guava, *Psidium guajava* L. (Myrtales: Myrtaceae), in the northern Espirito Santo State, Brazil. 146 specimens of mymarommatids were collected, of which 71, 55, 16, and 4 exemplars were obtained in the area cultivated with guava, papaya, citrus, and coconut, respectively. The mean numbers of mymarommatids collected in the period from April to June 2011 were significantly higher than those obtained in the other nine months. Two genera, *Mymaromma* and *Mymaromella* , were identified The most abundant genus was *Mymaromma* , comprising 93.8% of the total collection; however, the genus *Mymaromella* was encountered in all crops. This is the first record of the presence of mymarommatids in these agricultural systems.

## Introduction

Specimens of Mymarommatidae appear on all continents, but are rarely collected and are poorly represented in most collections, partly due to their small size ( Gibson et al. 2007 ). Studies made in northeast Montreal, Canada, reported only three specimens during a period of four months ( Clouâtre et al. 1989 ). In 1987, the studies indicated that slightly more than 200 specimens had been collected in the world ( Huber 1986 ); however, this number doubled when 32 exemplars were recorded in Venezuela (Garcia 2000) and 169 specimens were recorded in Brazil ( Bragança et al. 2004 ).

Members of the family Mymarommatidae are very small insects, slender, yellowish to light brown, with a body length ranging from 0.4 to 0.7 mm. They present certain distinctive features that allow them to be easily distinguished from other families of Hymenoptera ( Noyes and Valentine 1989 ; Gibson et al. 2007 ). The head shows the frontal and posterior surfaces joined by an inverted U-shaped arc of pleated membrane extending along the occiput from the base of each mandible, which allows the head to expand and contract in an accordion-like manner ( Gibson et al. 1999 ). The forewings are totally reticulated and spatulate with a fringe of long bustles, while the hindwings are reduced and apically bifurcate haltere-like. The metasomal abdominal petiole is two-segmented ( Gibson 1986 ; Gibson et al. 1999 ).

Both the hosts and the biology of Mymarommatidae are unknown. Some authors refer to their association with weevils in mosses ( Noyes 2003 ) or with bracket fungus ( Gibson 1993 ). Due to their small size, there are assumptions that they are parasitoids of eggs ( Yoshimoto 1984 ), possibly of arachnids ( Gibson 1986 ) or Psocoptera ( Huber et al. 2008 ).

Our study aimed to collect and identify families of parasitoids associated with agricultural systems located in northern Espirito Santo State, Brazil. The mymarommatids were obtained from material collected in yellow pan traps.

## Materials and Methods

### Study area

The surveys of mymarommatids were conducted in the municipalities of Linhares and Sooretama, which are situated in the northern region of Espirito Santo State, Brazil. In both localities, the climate is classified as hot, tropical, and dry, with dry winters (June–September) and rainy summers (December–March).

Linhares has an average annual rainfall of 1193 mm and mean temperatures of 19.6º C in winter and 32º C in summer. In Sooretama, the average annual rainfall is 1302 mm while the average annual temperature is 23° C.

### Data collection

Throughout one full year, insect collections were carried out using yellow pan traps. These traps consisted of a rectangular plastic bowl (39 cm x 29 cm x 6 cm) filled 1/3 of its capacity with water solution, formaldehyde, and few drops of detergent. After each sampling period, all the material was transported from the field to the laboratory, where the content of each bowl was passed through a fine mesh. The specimens of Mymarommatidae were sorted and maintained in 70% ethanol.

The traps were installed in two agricultural systems in the municipality of Linhares, one area cultivated with green dwarf coconut, *Cocos nucifera* L. (Arecales: Arecaceae) (19° 28' 9'' S, 40° 5' 37'' W, 28 m a.s.l.) and one area with guava, *Psidium guajava* L. (Myrtales: Myrtaceae) (19° 15' 36'' S, 40° 3' 26'' W, 30 m a.s.l.). In Sooretama, one of the areas was cultivated with papaya, *Carica papaya* L. (Brassicales: Caricaceae) (19° 7' 6'' S, 40° 4' 48'' W, 60 m a.s.l. ) and other with *Citrus* spp. L. (Sapindales: Rutaceae) (19° 7' 13'' S, 40° 5' 3'' W, 58 m a.s.l.).

**Figure 1.**
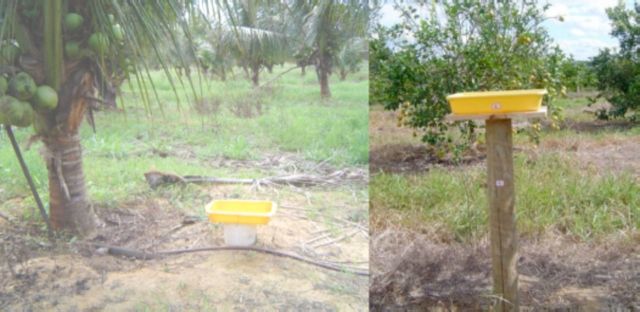
Yellow pan traps installed in the culture of *Cocos nucifera* on a plastic tube at a height of 0.2 meters above ground level (at left) and in the culture of *Citrus* spp. placed on wooden stakes at a height of 1.20 meters above ground level (at right). High quality figures are available online.

In the *C. nucifera* crops, 10 traps were placed beside the plants on plastic tubes at a height of 0.2 meters above ground level ( Figure 1 ) with a spacing of 25 m alternately in three rows. The collection and exchange of the trap solution was made weekly during 54 weeks, from June 2009 to July 2010.

In each area cultivated with *P. guajava* , *C. papaya* , and *Citrus* spp., 10 traps were installed. These traps were placed on wooden stakes at a height of 1.2 m above ground level ( Figure 1 ), spaced 25 meters from one another alternately in three rows. The collection and exchange of the trap solution was made weekly during 54 weeks, from June 2010 to July 2011.

In the Laboratory of Biological Control of Espirito Santo Rural Research and Extension Institute in Linhares, Espirito Santo State, Brazil, the exemplars of mymarommatids were separated into morphospecies and identified to the genus level according to keys of Gibson et al. (2007) . In the future, voucher specimens will be mounted and deposited in the collection of the Department of Ecology and Evolutionary Biology at the Federal University of Sao Carlos, Brazil.

### Statistical analysis

Statistical analyses were performed using Assistat Software version 7.6 beta ( www.assistat.com ). The data were analyzed by analysis of variance, and the mean numbers of mymarommatids obtained each month in the three crops *(P. guajava, C papaya* and *Citrus* spp.) were compared by the Scott-Knott test.

## Results

Throughout the study, a total of 146 specimens of Mymarommatidae were collected. Of these, 71 were collected from *P. guajava,* 55 from *C papaya,* and 16 from *Citrus* spp. In *C nucifera,* only 4 were collected during the entire period of samplings (2 specimens in September 2009; 1 in October 2009; 1 in March 2010).

The means (± SE) of mymarommatids obtained per week were: 1.06 ± 0.20 specimens in the culture of *C papaya,* 0.31 ± 0.01 specimens in *Citrus* spp., and 1.32 ± 0.28 in *P. guajava.*

**Figure 2.**
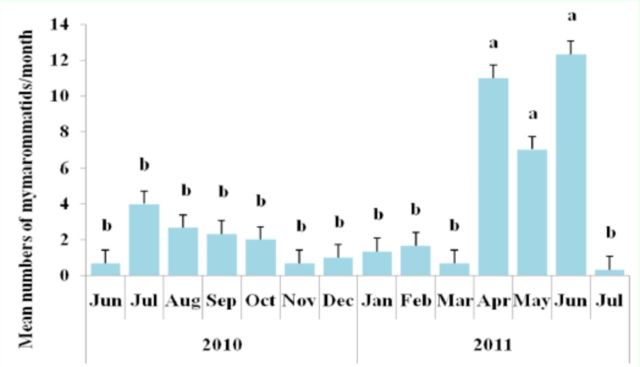
Mean numbers of Mymarommatidae collected/month in *Carica papaya, Citrus* spp., and *Psidium guajava* during June 2010 to July 2011 in Espirito Santo State, Brazil. Vertical bars with different letters above them are significantly different (ANOVA and Scott-Knott test, *p* < 0.01). Error bars ± SE. High quality figures are available online.

The differences between the mean numbers of mymarommatids collected during April to June 2011 and those obtained in the other months in *P. guajava, C papaya,* and *Citrus* spp. were highly significant (F = 3.77, *p* < 0.01). Data are shown in Figure 2 .

No Mymarommatidae were found in *Citrus* spp. in June 2010, August 2010, September 2010, November 2010, January 2011, March 2011, or July 2011. No mymarommatids were collected in *C. papaya* in June 2010, December 2010, March 2011, or July 2011. In *P. guajava* , none were collected in November 2010.

**Figure 3.**
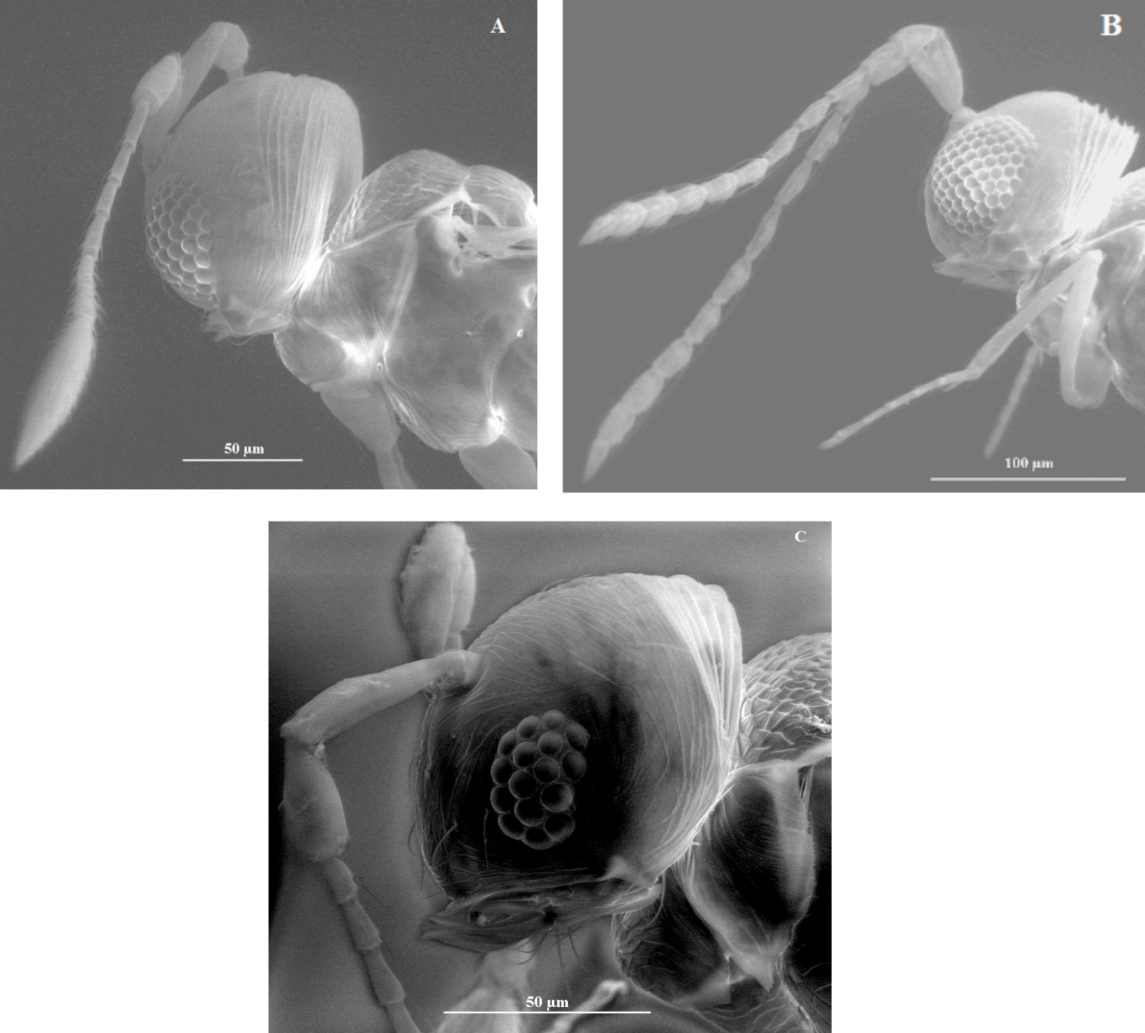
(A) Female and (B) male *Mymmaroma* sp. (C) Female *Mymaromella* sp. High quality figures are available online.

The results revealed the presence of two morphospecies belonging to two genera of Mymarommatidae, namely *Mymaromma* and *Mymaromella* ( Figure 3 ). The most abundant genus was *Mymaromma* , which comprised 93.8% of the total collection. *Mymaromella* sp. was encountered in all crops, and all individuals collected were females. The data are shown in Table 1 .

**Table 1.**
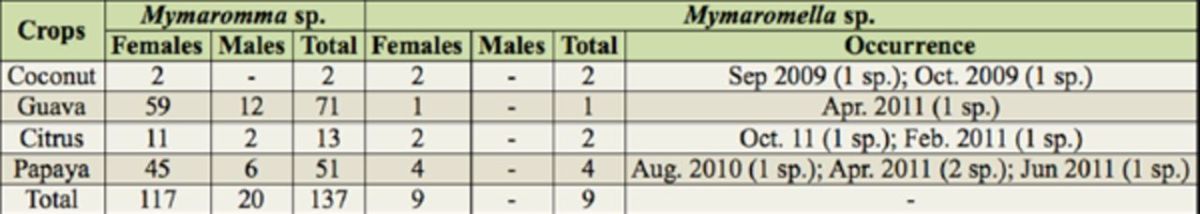
Total number of specimens from the genera *Mymaromma* and *Mymaromella* collected in coconut, guava, citrus, and papaya and period of occurrence and number of exemplars *of Mymaromella* sp. obtained in each culture in the Espirito Santo State, Brazil.

## Discussion

Most reports refer to the presence of Mymarommatidae in shady and relative moist areas, such as deciduous forests, including temperate regions of Europe and Canada, humid regions of Australia, and tropical forests of Indonesia ( Clouâtre et al. 1989 ; Noyes 1989 ). In the Neotropical region, the insect was found in Argentina, in the Misiones Province, in the locality of Loreto ( Fidalgo and De Santis 1982 ). In Brazil, it was found in the Cerrado of Sao Paulo State, in the Atlantic Forest of Espirito Santo and in the Cerrado of Tocantins State ( Penteado-Dias and Braga 2002 ; Azevedo et al. 2003 ; Bragança et al. 2004 ).

A review of the available literature revealed little information on the occurrence of these insects in agricultural systems. In Venezuela, yellow pan traps, interception, and Malaise traps allowed the collection of specimens of these insects in cocoa (Garcia 2000). On the island of the Azores, Moniz and Borges (2004) collected specimens in fig and chestnut. In Brazil, the only existing record was recorded in coffee crops in Bahia State ( Santos 2010 ).

Variation in insect abundance in tropical regions is a well-established fact, but little is known about the factors that determine this seasonality. In the tropics, variation of climate conditions can affect the seasonal patterns of insects ( Wolda and Fisk 1981 ). One of the most important factors in many regions is the change from the dry to the rainy season ( Wolda 1988 ). In this survey, the presence of a great number of specimens of Mymarommatidae was found in the dry season (April to June 2011). Similar results were recorded in Venezuela by Garcia (2000), who collected 16 specimens of mymarommatids in the dry season (February– March 1999), 10 specimens at the beginning of the rainy season (May), and 3 during the rainy season (July–August) of 1999. In southeastern Quebec, the active period of Mymarommatidae is from June to August ( Clouâtre et al. 1989 ).

However, the seasonal abundance of insects cannot be explained only by one or a group of climate factors. Many other factors, such as interspecific competition, parasitism, predation, distribution of a food resource at a particular time of the year, and habitat variables, among others, appear to act together with climate factors to mold the patterns of distribution and abundance of insects.

According to Noyes (1989) , it is possible that larger Hymenoptera are attracted by the water in the trap, especially in dry climates, and that may explain why a greater number of specimens of mymarommatids were collected in the dry season.

It is known that heavy rain can have a depressant effect on the flight activity of some species, and light rain may increase the activity of others (Lewis 1965). Furthermore, heavy rainfall can kill insects that live in the soil by flooding. Huber et al. (2008) postulated that *M. palella* probably parasitizes hosts in soil or litter because it is the only described species of *Mymaromella* that is adapted to crawling, as evidenced by the lack of ocelli and relatively few ommatidia in the eyes. In this survey, it was not possible to identify the species of *Mymaromella,* but the morphospecies caught had the features cited above ( Figure 3C ). So, considering the possibility that its habitat is the soil, the rainy period may have negatively affected the population of *Mymaromella.*

Although many authors consider it possible that mymarommatids are parasitoids of eggs due their small size and their very short ovipositors, in this study it was not possible to provide any evidence for the hosts of these insects. Therefore, it would be necessary to collect hosts in the plants and transport them to the laboratory to observe the emergence of parasitoids.

These findings add to the previous surveys conducted in Brazil ( Penteado-Dias and Braga 2002 ; Azevedo et al. 2003 ; Bragança et al. 2004 ; Santos 2010 ), resulting in 319 mymarommatids collected in this country. Our study reports for the first time the family Mymarommatidae in crops of *C. nucifera, C. papaya, Citrus* spp., *and P. guajava.*
